# 648. Cefiderocol as Rescue Therapy for *Pseudomonas aeruginosa* and Other Difficult-To-Treat Gram-Negative Infections

**DOI:** 10.1093/ofid/ofac492.700

**Published:** 2022-12-15

**Authors:** Andrew Chou, David J Ramsey, Barbara Trautner

**Affiliations:** Michael E. DeBakey Veterans Affairs Medical Center, Houston, Texas; Michael E. DeBakey Veterans Affairs Medical Center, Houston, Texas; Michael E. DeBakey Veterans Affairs Medical Center / Baylor College of Medicine, Houston, TX

## Abstract

**Background:**

Cefiderocol is a novel siderophore cephalosporin and the only beta-lactam that has demonstrated *in vitro* activity against metallo-beta-lactamase producing organisms. The open-label phase 3 study CREDIBLE-CR unexpectedly found a higher mortality rate in the cefiderocol group compared to best available therapy. Post-approval, real-world clinical data of cefiderocol use are limited. We sought to describe nationwide, real-world outcomes, clinical characteristics, and microbiological characteristics of infections treated with cefiderocol.

Cefiderocol use since FDA approval (Nov 2019)

**Figure:**
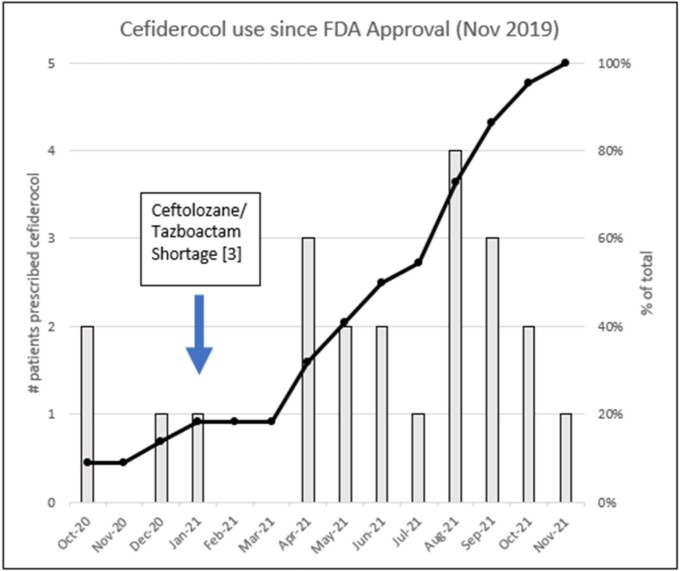

**Methods:**

The Department of Veterans Affairs (VA) Corporate Data Warehouse was queried to identify any patient prescribed cefiderocol at any of the 171 VA medical centers from date of FDA approval in 11/2019 until 11/2021. Clinical, pharmacy, demographics, and microbiology data were computationally extracted, supplemented by manual review. All patient charts were manually reviewed to determine the indication for cefiderocol and clinical outcomes.

**Results:**

Twenty-five patients have been prescribed cefiderocol; two received only one dose, and one patient had incomplete records. Prior to 2021 there were four prescription, and since 2021 there have been 21 prescriptions. Of the 22 evaluated patients, their median age was 70 years and 82% were receiving care in the ICU. Infecting organisms (n=25) were *Pseudomonas aeruginosa* 63%, *A. baumannii* 18.5%, and Enterobacterales 18.5%. Cefiderocol susceptibility was tested on 17 isolates, of which 15 (88.2%) were susceptible. The two non-susceptible organisms were *A. baumannii* and *P. aeruginosa*. The susceptibilities of the 25 strains overall were as follows: carbapenems (13.8%), ceftazidime/avibactam (4 of 12, or 33.3%), and ceftolozane/tazobactam (7 of 15, or 46.7%). The most common infectious syndromes were pulmonary and central line-related infections, occurring in 38.5% and 23.1%, respectively. The 28-day mortality rate was 31.8%, 28-day clinical failure rate was 45.5%, and the 28-day microbiological failure rate was 31.8%.

**Conclusion:**

Patients prescribed cefiderocol in this series had severe infections with high rates of mortality and few alternative antibiotic options. Susceptibility to cefiderocol was higher than for other antibiotics tested.

**Disclosures:**

**Barbara Trautner, MD, PhD**, Genetech: Advisor/Consultant.

